# Ambient effects on electrical characteristics of CVD-grown monolayer MoS_2_ field-effect transistors

**DOI:** 10.1038/s41598-017-04350-z

**Published:** 2017-06-22

**Authors:** Jae-Hyuk Ahn, William M. Parkin, Carl H. Naylor, A. T. Charlie Johnson, Marija Drndić

**Affiliations:** 1 Department of Physics and Astronomy, University of Pennsylvania, Philadelphia, Pennsylvania 19104 United States; 2 Department of Electronic Engineering, Kwangwoon University, Seoul, 01897 South Korea

## Abstract

Monolayer materials are sensitive to their environment because all of the atoms are at their surface. We investigate how exposure to the environment affects the electrical properties of CVD-grown monolayer MoS_2_ by monitoring electrical parameters of MoS_2_ field-effect transistors as their environment is changed from atmosphere to high vacuum. The mobility increases and contact resistance decreases simultaneously as either the pressure is reduced or the sample is annealed in vacuum. We see a previously unobserved, non-monotonic change in threshold voltage with decreasing pressure. This result could be explained by charge transfer on the MoS_2_ channel and Schottky contact formation due to adsorbates at the interface between the gold contacts and MoS_2_. Additionally, from our electrical measurements it is plausible to infer that at room temperature and pressure water and oxygen molecules adsorbed on the surface act as interface traps and scattering centers with a density of several 10^12^ cm^−2^ eV^−1^, degrading the electrical properties of monolayer MoS_2_.

## Introduction

Two-dimensional transition metal dichalcogenides (TMDCs) have received significant attention due to a wide range of unique physical and electronic properties^1–3^. TMDCs have a layered structure of the form X-M-X, where M is a transition metal and X is a chalcogen, with strong intralayer covalent bonds and weak interlayer van der Waals interaction allowing their exfoliation into monolayers. Monolayer MoS_2_ is especially attractive for applications in electronics and optoelectronics^1–6^. It is a promising channel material for field-effect transistors (FETs) because of a large bandgap for effective switching with a high on/off ratio and low power operation, a strong electrostatic control of the channel potential due to atomic thickness, and high mobility potentially allowing fast operation^4, 5^. In addition, the direct bandgap in monolayer MoS_2_ allows for a high optical absorption coefficient and efficient carrier excitation suitable for optoelectronic applications^6^. Furthermore, the atomic thickness and mechanical robustness of monolayer MoS_2_ enable other applications such as flexible and transparent electronics^7^.

Monolayer MoS_2_ samples can be obtained from bulk crystals by a micromechanical cleavage technique developed for the production of graphene^8^. To overcome limitations of this method in precise control of sample thickness and size required for practical application, synthesis methods based on chemical vapor deposition (CVD) have been developed for uniform and large-area production of monolayer MoS_2_^9, 10^. FETs fabricated with CVD-grown monolayer MoS_2_ show device performance with mobility of ~4–45 cm^2^/Vs and on-off ratio of >10^6^ under vacuum at room temperature^10–14^. The electrical properties of such atomically thin semiconducting channels are sensitive to ambient gases (*i.e*., water and oxygen molecules) because almost all atoms are located on the surface, which in turn can be utilized as sensitive chemical and gas sensors^15, 16^. Previous studies have shown that the adsorbed gases on the MoS_2_ channel result in degradation of device conductance^17, 18^, hysteresis^19–21^, and electrical stress-induced threshold voltage instability^22^. It also has been reported that vacuum annealing increases the device conductance by desorbing the gas molecules from the MoS_2_ samples^13, 23, 24^. Although the previous reports shed light on the importance of ambient gases in device performance, some of the mechanisms behind ambient-related effects still remain to be studied in more detail: i) the mechanisms governing the changes observed in the electrical parameters under ambient pressure changes, ii) the origins of improved electrical properties under vacuum annealing, iii) the understanding of the role of interface traps and their formation as the ambient gases are introduced, and iv) the role and nature of electrical contacts to the MoS_2_ layer.

The goal of this work is to answer the aforementioned mechanism-related questions more systematically, thus contributing to a better understanding of the ambient effects on the performance of MoS_2_ FET devices and critically comparing with previously published results^17–22^. In this work, we studied the electrical parameters of MoS_2_ transistors including field-effect mobility, threshold voltage, hysteresis, and subthreshold slope, and their dependence on pressure. We fabricated unencapsulated MoS_2_ FETs (a total of 13 devices) consisting of the FET channel and a back-gate, exhibiting similar device performance to other devices fabricated in previous works^10–13^ with mobility of 11.3 ± 0.9 cm^2^/Vs, on/off ratio of >10^6^, and contact resistance of 155 ± 30 Ω mm under vacuum at room temperature. We monitor changes in the electrical parameters of the MoS_2_ FETs under different vacuum pressures. Based on these measurements, we discuss the likely underlying charge transfer mechanism governing the MoS_2_ channel properties and the modification of MoS_2_-metal contact nature from Schottky to ohmic contacts in the absence of adsorbed gas molecules. We can extract the interface trap density from these measurements in order to quantify the equivalent density of scattering centers created on the MoS_2_ surface due to adsorbed gas molecules. Moreover, we carefully analyze the effects of vacuum annealing on mobility and contact resistance of the MoS_2_ FETs using a transfer length method (TLM) measurement^25^, which is a widely used technique for the extraction and analysis of contract resistance in typical FETs including MoS_2_ FETs^26^ by measuring the resistances between each pair of metal contacts separated by various distances. This study provides a more comprehensive analysis of the interaction between ambient gases and MoS_2_ FETs than previously considered and helps guide the future design of high-performance MoS_2_ FETs and for highly sensitive MoS_2_-based chemical and gas sensors.

## Results and Discussion

**Figure 1:**
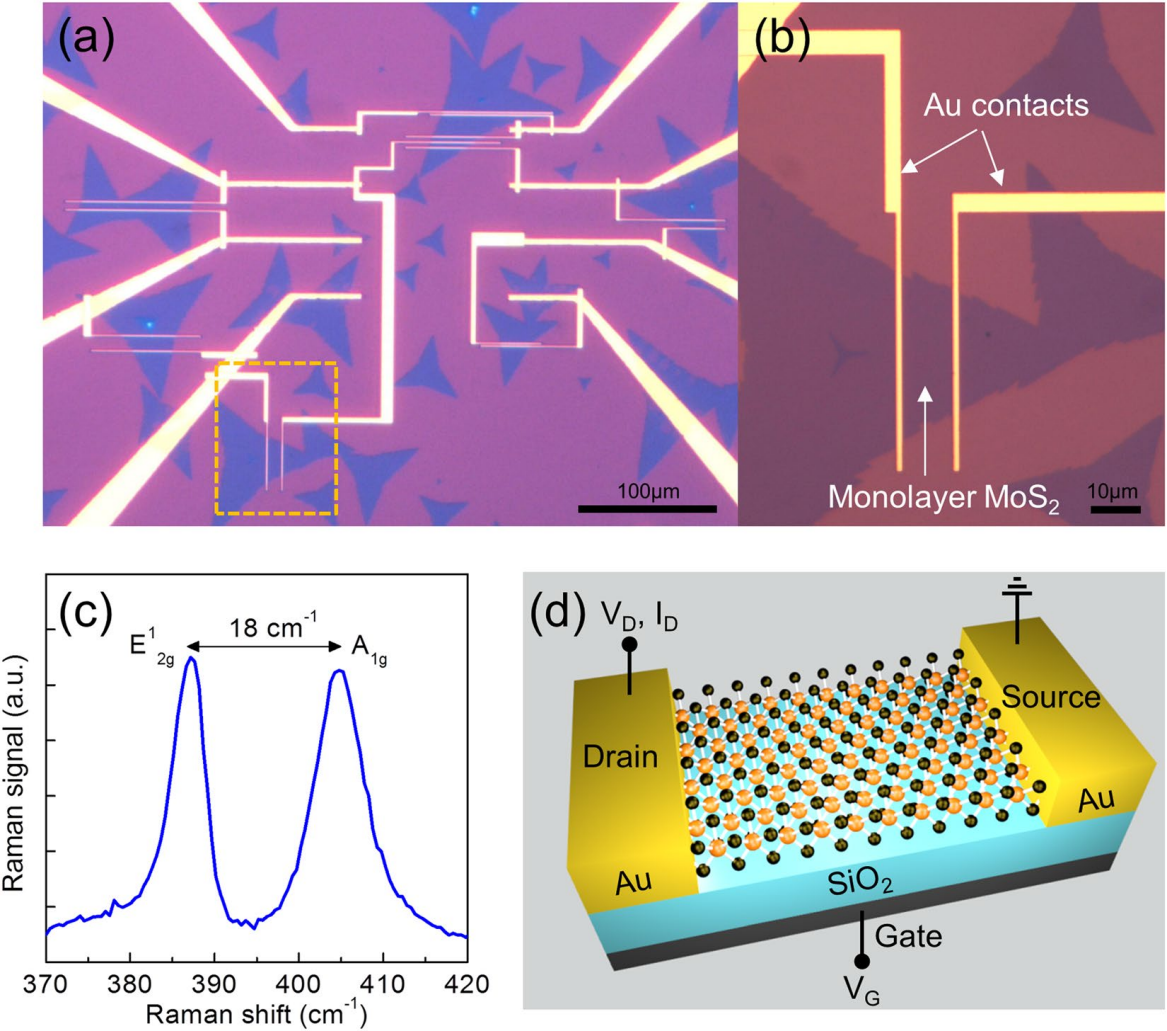
Fabrication of CVD-grown monolayer MoS_2_ FETs. (**a,b**) Optical images of the monolayer MoS_2_ FETs. The magnified view in (**b**) shows a monolayer MoS_2_ flake with Au contacts on top. (**c**) Raman spectrum of the monolayer MoS_2_ flake with a peak spacing of 18 cm^−1^ between the E^1^_2g_ and A_1g_ vibrational modes, confirming the monolayer nature of the CVD-grown MoS_2_^28^. (**d**) Three-dimensional schematic of the monolayer MoS_2_ FET with measurement configuration. The monolayer MoS_2_ flake is placed on a heavily doped p-type silicon substrate with a 285 nm-thick SiO_2_ layer for back-gate operation.

We fabricated back-gated monolayer MoS_2_ FETs using standard nanofabrication processes including photolithography, electron-beam lithography, and metal deposition (Fig. 1a,b,d). Details of the MoS_2_ growth and device fabrication are presented in Methods. Briefly, monolayer MoS_2_ flakes were grown on a SiO_2_/Si substrate by CVD using a previously published procedure^27^. As shown in Fig. 1c, the monolayer thickness of the CVD-grown MoS_2_ flakes is confirmed by its Raman spectrum where the frequency difference between E^1^_2g_ and A_1g_ modes (~18 cm^−1^) indicates the monolayer nature of MoS_2_^28^. 100-nm thick Au electrodes were contacted to the individual MoS_2_ flakes by electron-beam lithography, thermal metal evaporation, and a lift-off process. For contacts, we used only Au without any adhesion layers (such as the typically used Cr or Ti adhesion layers) to reduce the contact resistance, as was demonstrated previously^4^. A gentle lift-off process in acetone solution enabled good adhesion of the Au layer onto the device surface without peeling off. The MoS_2_ FETs have channel widths of 12 to 80 μm and channel lengths of 1 to 10 μm. To investigate ambient effects on the electrical characteristics of monolayer MoS_2_ FETs, we measured the drain current of the fabricated devices using Keithley source meters (Keithley 2400 and 2410) as a function of gate voltage using a sweep rate of 5 V/s inside a vacuum probe station (Lakeshore Cryogenics).

**Figure 2:**
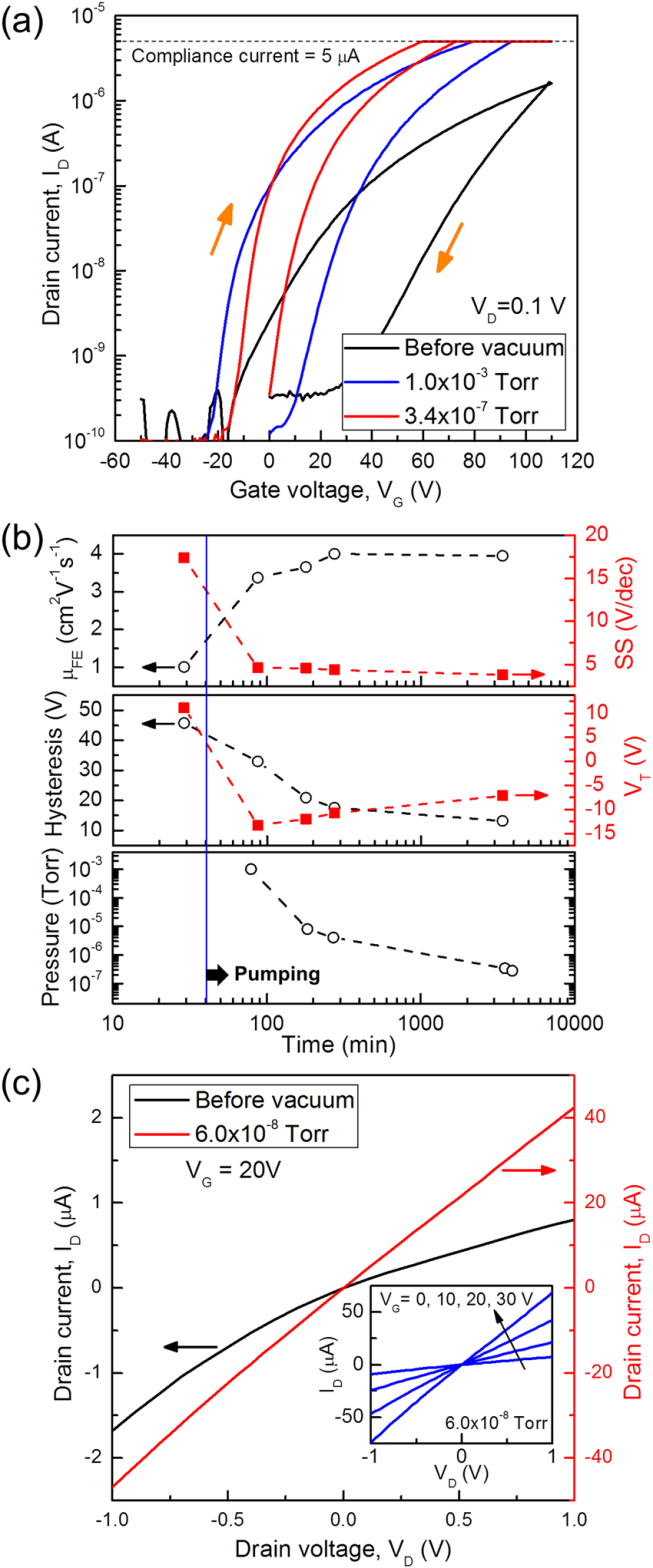
Dependence of the electrical characteristics of the monolayer MoS_2_ FETs on ambient pressure. (**a**) Drain current (*I*_*D*_) of the monolayer MoS_2_ FET as a function of gate voltage (*V*_*G*_) under a constant drain voltage (*V*_*D*_) of 0.1 V with different ambient pressure from atmospheric pressure to 1.0 × 10^−3^ Torr and 3.4 × 10^−7^ Torr. The drain current of the device is measured inside a vacuum probe station as the gate voltage is swept from −50 V to 110 V and then back to 0 V with the sweeping direction indicated by orange arrows and the sweeping rate at 5 V/s. We set the compliance current to 5 μA for the drain current to improve the measurement resolution near the subthreshold regime. (**b**) Continuous measurement of electrical parameters as the pressure is gradually lowered from ambient to 3.4 × 10^−7^ Torr: hysteresis, threshold voltage (*V*_*T*_), field-effect mobility (*μ*_*FE*_), and subthreshold swing (*SS*) – defined in the main text. A non-monotonic dependence of threshold voltage on pressure is observed in the graph of *V*_*T*_ vs. time (middle panel). The dashed lines through the data points are guides to the eye. (**c**) The *I*_*D*_ − *V*_*D*_ characteristics of the monolayer MoS_2_ FET before and after vacuum (6.0 × 10^−8^ Torr). Inset: output characteristics of the MoS_2_ device under vacuum with various gate voltages. The linear *I*_*D*_ − *V*_*D*_ characteristics indicate that the contact is ohmic.

As-fabricated monolayer MoS_2_ FETs show n-type behavior as shown in Fig. 2a. As the gate voltage (*V*_*G*_) is swept from −50 V to 110 V with a constant drain voltage (*V*_*D*_) of 0.1 V, the drain current (*I*_D_) increases from the off-state current of ~100 pA to the on-state current of ~1.6 μA. We observe a few regimes shown in typical FETs that can be defined as follows: i) *the off-state regime* (*V*_*G*_ < −20 V) where the minimum or negligible drain current flows due to the high MoS_2_ channel resistance, ii) *the subthreshold regime* (*V*_*G*_ = −20 to ~20 V) where the drain current is an exponential function of the gate voltage, showing an approximately linear behavior of the log(*I*_*D*_) vs. *V*_*G*_ curve, and iii) *the on-state regime* (*V*_*G*_ > 20 V) where the MoS_2_ FET is “turned on” and the log(*I*_*D*_) vs. *V*_*G*_ curve is no longer linear. Here, the threshold voltage (*V*_*T*_) that requires to “turn on” the devices is defined as the gate voltage at the drain current of 10 nA.

Because the number of water and oxygen molecules per unit volume is reduced under vacuum, we can investigate the ambient effect on electrical characteristics of the MoS_2_ FETs by reducing the ambient pressure. As the ambient pressure was reduced, the on-state current increased and eventually reached the compliance current limit of the Keithley source meter (Fig. 2a). The field-effect mobility (*μ*_FE_) can be extracted from the following equation^29^:

$${\mu }_{FE}=\frac{1}{{C}_{ox}}\frac{L}{W}\frac{d{I}_{D}}{d{V}_{G}}\frac{1}{{V}_{D}}$$

where *C*_*ox*_ is the capacitance per unit area of 285 nm-thick SiO_2_ (12.1 nF/cm^2^), *L* is the channel length (~1 μm), and *W* is the channel width (~32 μm). The field-effect mobility is increased about 4 times from 1.0 cm^2^/Vs under atmospheric pressure to 4.0 cm^2^/Vs at 3.4 × 10^−7^ Torr (Fig. 2b). This result is consistent with previous results showing that the conductance of the MoS_2_ FETs is increased under vacuum or low humidity conditions^17–19^. We continuously monitored the trend of the field-effect mobility as a function of time with decreasing pressure. In this fashion, we were able to observe the changes in the field-effect mobility gradually for a wide range of pressures from atmospheric pressure to 3.4 × 10^−7^ Torr. Indeed, the gradual increase of the field-effect mobility was observed as shown in Fig. 2b. From this we conclude that the field-effect mobility of the monolayer MoS_2_ FETs is monotonically enhanced in high vacuum where the density of water and oxygen molecules decreases.

In Fig. 2b we plot the dependence of the threshold voltage *V*_*T*_, with decreasing pressure. Interestingly, unlike the monotonic change in the field-effect mobility, the threshold voltage first decreases with decreasing pressure down to a minimum value, after which it increases as the pressure is lowered further (Fig. 2b). This non-monotonic feature of the threshold voltage is reproducible and seen in multiple devices (Fig. S1). To the best of our knowledge, this non-monotonic dependence of *V*_*T*_ vs. pressure has not been reported in previous papers studying ambient effects on MoS_2_ FETs^17–21^.

Previous works have hypothesized that the threshold voltage under vacuum is related to the water and oxygen molecules that can capture the electrons from the MoS_2_ channel^13, 18, 23^. Density functional theory calculations revealed that approximately 0.04 electrons per O_2_ and 0.01 electrons per H_2_O are transferred to the molecules, depleting the MoS_2_ channel^30^. When a negative gate bias is applied, the captured electrons in the adsorbates can be donated back to the channel. Thus, the threshold voltage should be low when the adsorbate concentration is high because in this case a lower gate voltage is required to reach the on-state regime in the MoS_2_ channel. If the charge transfer between the MoS_2_ channel and the water and oxygen adsorbates is the only factor to determine the threshold voltage, we should see a continuous increase in the threshold voltage as the pressure is reduced. However, the threshold voltage initially decreases as the pressure is lowered. Therefore, in addition to any channel doping there is a competing factor leading to a decreasing threshold voltage as the pressure is reduced. This motivates careful consideration of additional reasons behind the decrease in the threshold voltage.

Specifically, another possible reason for the reduced threshold voltage under vacuum conditions that has not been described in previous reports is the change of the nature of the contacts between MoS_2_ and metal electrodes caused by adsorbed gases. In particular, as the adsorbed molecules near MoS_2_-metal electrodes are desorbed, the contacts are changed from Schottky contacts to ohmic contacts. This change of contacts is indicated by the transition of the nonlinear to the linear *I*_*D*_ − *V*_*D*_ characteristics under vacuum (6.0 × 10^−8^ Torr) as shown in Fig. 2c. Furthermore, this change of contact properties caused by desorbed gases would result in the reduction of threshold voltage. This is because at the ohmic contacts, a higher injection of electrons from metal contacts to the MoS_2_ channel allows the device to turn on at a lower gate voltage. Compared to the ohmic-contacted MoS_2_ FETs with Au contact (100 nm), Schottky-contacted devices with Ti/Au contacts (3 nm/100 nm) show a larger decrease in the threshold voltage after removal of adsorbed molecules with thermal annealing in vacuum (Fig. S2). This indicates that desorption of molecules near the contacts may be the dominant factor in the initial reduction of the threshold voltage by lowering the Schottky barriers and improving charge injection. In addition, Schottky contacts are more sensitive to the gas molecules than ohmic contacts, and can be utilized to fabricate highly sensitive gas sensors^16^.

The threshold voltage starts to increase again from −13 V to −7 V as the ambient pressure further decreases from 1.0 × 10^−3^ Torr to 3.4 × 10^−7^ Torr (Fig. 2b). This feature is related to the release of the captured electrons from the water and oxygen molecules into the MoS_2_ channel at negative gate bias near the off-state regime^20, 22^, resulting in a visible hysteresis. Under high vacuum conditions, the lower trap density reduces the number of trapped carriers that can be donated back to the channel when a negative gate voltage is applied, resulting in a decreased drain current and an increased threshold voltage (Fig. S3). This decrease of the charge transfer can explain the reduced hysteresis under vacuum conditions (Fig. 2a,b). Our experimental finding that the water and oxygen molecules in direct contact with the MoS_2_ channel are the major source of hysteresis is further supported by additional measurements with top-gated monolayer MoS_2_ FETs with an added Al_2_O_3_ dielectric, where we observed a negligible hysteresis even in atmosphere condition due to the passivation of the surface (Fig. S4).

To improve device performance with low power operation and fast switching between the off- and on-state regimes, it is important to increase the gate control over the drain current in the subthreshold regime and thus reduce the subthreshold slope (SS), defined as $\frac{d{V}_{G}}{d\,\mathrm{log}({I}_{D})}$ in the subthreshold regime. In our measurements we observe that the subthreshold slope is enhanced by high vacuum from 17.4 V/dec (before vacuum) to 3.8 V/dec (pressure = 3.4 × 10^−7^ Torr) (Fig. 2b). The value of the subthreshold slope of typical FETs is decreased with lower density of interface traps in the channel for a given device size^29^. In our case, interface traps on the MoS_2_ channel can be produced by water and oxygen molecules in the ambient air. The lower density of these interface traps under high vacuum compared to ambient conditions can explain the observed reduction in the value of the subthreshold slope. For fast switching and low power operation of the MoS_2_ FETs, it is therefore important to control and reduce the traps caused by the adsorbed water and oxygen molecules.

**Figure 3:**
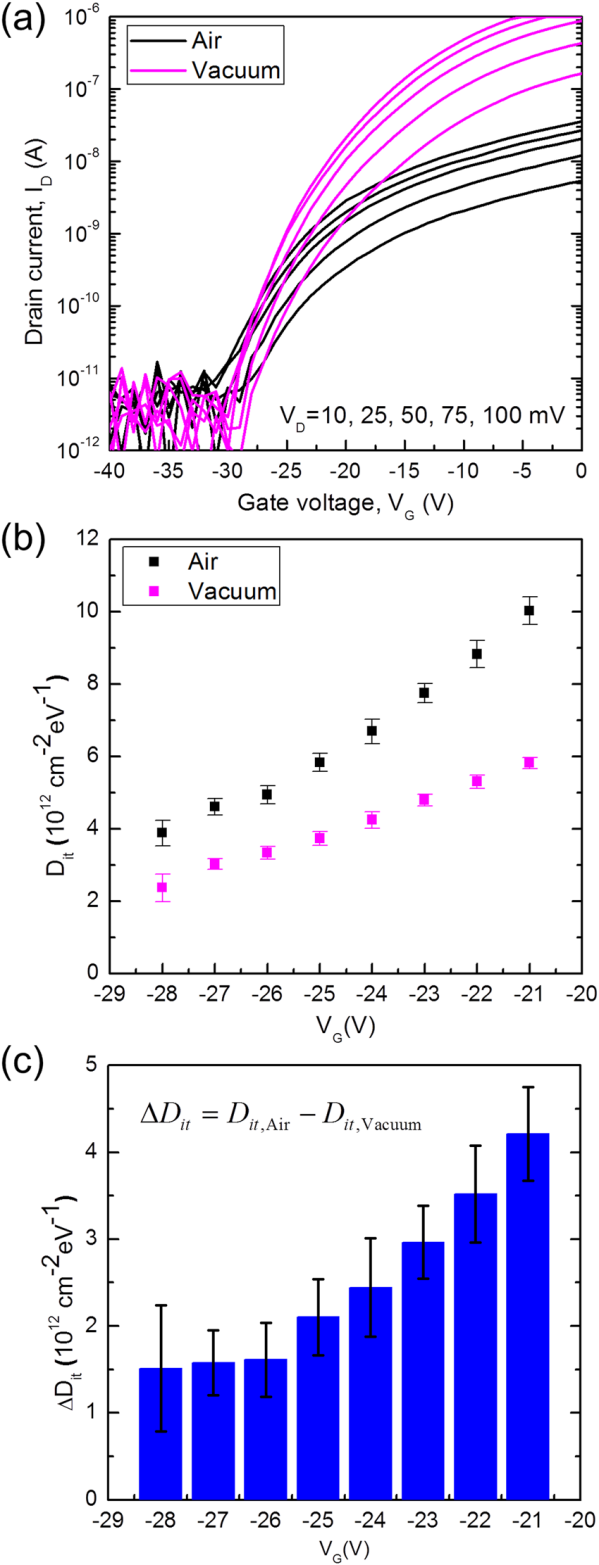
Extraction of interface traps density under different ambient conditions. (**a**) Measurement of the drain current (*I*_*D*_) as a function of gate voltage (*V*_*G*_) with different values of the drain voltage (*V*_*D*_) to extract the energy distribution of interface traps that can explain the data. The drain voltage is modulated with the values of 10, 25, 50, 75 and 100 mV. The same device is measured under two ambient conditions of “Air” for atmospheric pressure and “Vacuum” for vacuum condition (4.0 × 10^−8^ Torr) after vacuum annealing (400 K for ~14 hr). (**b**) Extracted interface trap density for the two ambient conditions using a subthreshold slope method, described in Methods. (**c**) Difference of the interface trap density between the two ambient conditions (Δ*D*_*it*_ = *D*_*it*_,_Air_ − *D*_*it*_,_Vacuum_, where *D*_*it*_,_Air_ and *D*_*it*_,_Vacuum_ are the interface trap densities under atmospheric pressure and vacuum condition (4.0 × 10^−8^ Torr) after vacuum annealing (400 K for ~14 hr), respectively).

We can quantitatively analyze the interface traps produced by the water and oxygen molecules by extracting the interface trap density (*D*_*it*_) of the monolayer MoS_2_ FETs according to different ambient conditions. Details of the extraction of interface trap density from the *I*_*D*_ − *V*_*D*_ characteristics in Fig. 3a are given in Methods. As shown in Fig. 3b, the interface trap density is lowered in vacuum, which confirms that water and oxygen molecules create the interface traps on the MoS_2_ channel as previously discussed. From the data in Fig. 3c, we would expect that the interface trap density caused by the adsorbed molecules is within the order of 10^12^ cm^−2^ eV^−1^. Our further analysis suggests that the adsorbed water and oxygen molecules help create acceptor-type traps^29^ that become negatively charged in a such way that the trap energy levels below the Fermi level are filled by electrons and that these interface traps are shallow traps^29^ where the energy states are mainly located near the conduction band (See Supplementary information).

For the interface trap density, the other major factor to be considered is the role of defects. CVD-grown MoS_2_ monolayers have intrinsic structural defects due to the imperfection of the growth process^31^. When the MoS_2_ sample is exposed to the ambient environment, the adsorption of water and oxygen molecules is preferred at the defect sites with higher binding energy^30, 32^. This implies that a higher defect density increases the number of adsorbates and results in a higher interface trap density. Because the adsorbates make stronger bonds at the defect sites, thermal annealing at high temperature is required to remove adsorbates^17^. Therefore, controlling intrinsic defects during the material growth is important to reduce the interface trap density produced by the adsorbed water and oxygen molecules.

**Figure 4:**
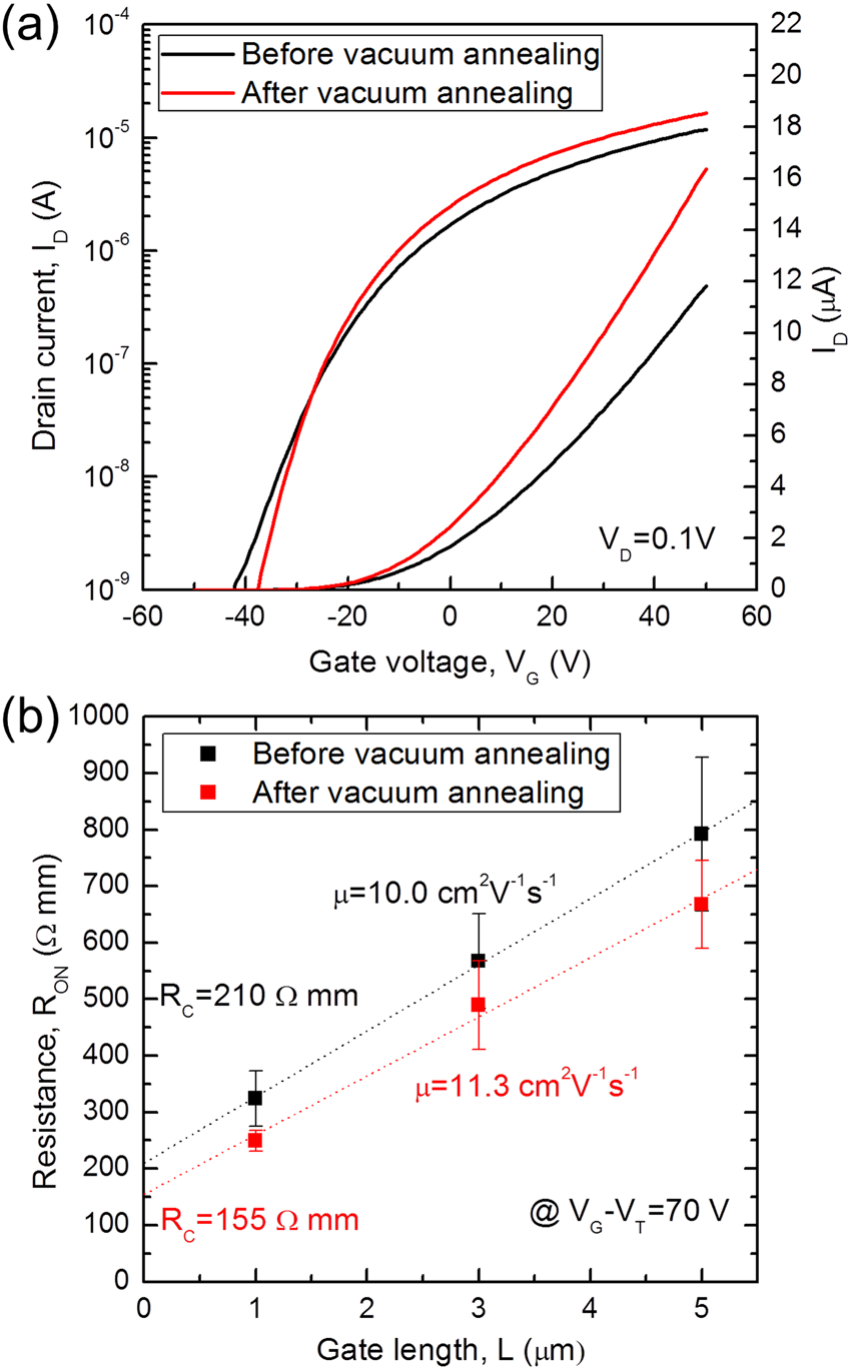
Effects of vacuum annealing on the electrical characteristics of the monolayer MoS_2_ FETs. (**a**) Drain current (*I*_*D*_) of the monolayer MoS_2_ FET as a function of gate voltage (*V*_*G*_) before and after vacuum annealing (400 K for ~14 hr), measured under vacuum (~6.0 × 10^−8^ Torr). (**b**) Contact resistance (*R*_*C*_) and mobility (*μ*) before and after vacuum annealing. For a fair comparison, the same gate voltage in excess of the threshold voltage (*V*_*G*_ – *V*_*T*_ = 70 V) is applied for the both conditions. The errors were calculated from total 13 devices with at least three devices at a given channel length (*L*).

In addition to the pressure, we have also investigated the effects of thermal annealing in vacuum. This was motivated by previous works that showed vacuum annealing to be an effective method to remove water and oxygen molecules bound on the MoS_2_ surface^13, 17, 23, 24, 30^. For an accurate analysis of the annealing effects, we employed a TLM measurement^25, 26^, which is the standard method for the extraction of contact resistance in typical FETs by fitting the on-state resistance as a function of channel length. Details of the TLM measurement are presented in Methods. We find that vacuum annealing at 400 K for ~12 hr led to the reduction of the contact resistance (*R*_*C*_) by ~26% from 210 ± 9 Ω mm to 155 ± 30 Ω mm and the increase of the mobility (*μ*) by ~13% from 10.0 ± 0.2 cm^2^/Vs to 11.3 ± 0.9 cm^2^/Vs and (Fig. 4b). These experimental results provide the direct answer for the origins of the improved conductance of the MoS_2_ FETs after vacuum annealing as observed in Fig. 4a: the decreased MoS_2_-metal contact resistance and the increased mobility of the MoS_2_ channel itself. The reduced adsorbates by vacuum annealing can lower the Schottky barriers formed between the MoS_2_ and metal contacts, resulting in lower contact resistance. In addition, vacuum annealing can reduce the density of adsorbates on the MoS_2_ channel surface which serve as interface traps and thus it leads to the increased mobility. Further comments on the effects of vacuum annealing are provided in Supplementary information.

## Conclusions

In summary, we investigated ambient effects (*i*.*e*., pressure and temperature) on the electrical characteristics of CVD-grown monolayer MoS_2_ FETs. As the measurement environment of the monolayer MoS_2_ FETs was changed from normal pressure to high vacuum (~10^−7^ Torr), we observed the gradual improvement of the electrical properties including increased field-effect mobility, reduced hysteresis, and decreased subthreshold slope. This result confirmed that the water and oxygen molecules adsorbed on monolayer MoS_2_ FETs degrade the device performance, as expected. We observed and analyzed the non-monotonic change in the threshold voltage with decreasing vacuum pressure and suggested the major factors to determine the threshold voltage: i) the adsorbate-induced metal-MoS_2_ contacts change from Ohmic to Schottky, and, ii) the charge transfer between the adsorbed gas molecules and the MoS_2_ channel. We also infer from our measurements that it is likely that water and oxygen molecules serve as acceptor-type, shallow traps with a level of several 10^12^ cm^−2^ eV^−1^ based on extraction of the interface trap density using a subthreshold slope method. We showed that the conductance is improved not only by reduced contact resistance between MoS_2_ and metal electrodes but also by the increased mobility of the MoS_2_ channel itself. A better understanding of all the various possible mechanisms in play due to the interaction between the atmospheric molecules and the MoS_2_ channel and MoS_2_-Au gold contacts on the electrical characteristics of the MoS_2_ FETs will be used to design high-performance nanoelectronics and chemical sensors based on two-dimensional transition metal dichalcogenides where surfaces govern the electrical properties. Finally, the analysis approach presented here to extract the interface trap density, mobility, and contact resistance may be useful for other similar studies analyzing the electrical behavior of unencapsulated two-dimensional devices under various ambient conditions, including the presence of gas molecules in gas sensor applications.

## Methods

### Growth of monolayer MoS_2_ flakes

Monolayer MoS_2_ flakes were grown directly on a Si/SiO_2_ growth substrate by chemical vapor deposition (CVD). A growth promoter (1% sodium cholate) is spin coated on the substrate at 3000 rpm for 60 sec and micro-droplets of a saturated solution of ammonium heptamolybdate are applied to the corners of the substrate. The substrate is then inserted into the center of a 1inch CVD furnace; it is accompanied with 25 mg of Sulfur that will be placed at a distance of 17 cm upstream. The furnace is ramped up to 700 °C at a rate of 70 °C/min under 500 sccm N_2_. After a 25 min growth time, the furnace is rapidly cooled and monolayer MoS_2_ flakes have grown across the substrate.

### Fabrication of MoS_2_ field-effect transistors

A highly p-doped Si wafer with a 285 nm-thick SiO_2_ layer on top was used as a back-gate substrate. Large electrodes with a 5 nm-thick Cr and a 80 nm-thick Au layer were fabricated using a photolithography tool (SUSS MicroTec MA6 Gen3 Mask Aligner), a custom-built thermal evaporator, and a lift-off of the photoresist in acetone overnight. A layer of PMMA resist (495 PMMA C4) with a thickness of ~300 nm was coated on the MoS_2_ substrate with spin-coating at 4000 rpm for 45 sec. The PMMA layer was cut into small rectangles (~5 mm × 5 mm) and the MoS_2_ substrate was floated on a KOH bath (56 mg/mL). After the substrate sank to the bottom of the KOH bath, each piece of PMMA/MoS_2_ flakes was scooped with a polyethylene terephthalate (PET) film and transferred to deionized water. After repeating this process at least three times to remove the residual KOH solution, we transferred the PMMA/MoS_2_ samples to the pre-patterned chips with large electrodes and dried at room temperature for overnight and then removed the PMMA layer on top in acetone solution for overnight.

We prepared a CAD file for small electrodes to connect the MoS_2_ flakes and large electrodes by importing optical images of the MoS_2_ flakes on the pre-patterned chip. The patterns for the small electrodes in the CAD file were transferred to the PMMA resist with an electron-beam lithography system (Elionix ELS-7500EX) at beam energy of 50 kV, beam current of 100 pA, and dose 350 μC/cm^2^, followed by development of the exposed resist in IPA:MIBK (3:1) solution for 1 min. The Au electrodes were contacted to the monolayer MoS_2_ flakes with metal deposition of 100 nm-thick Au at pressure below 3 × 10^−6^ Torr using a thermal evaporator (Lesker PVD75) and lift-off process in acetone solution overnight. For stable back-gate measurement, we deposited a 1.5 nm-thick Cr and a 60 nm-thick Au layer as a back-gate electrode onto the back-side of the chip using a custom-built metal evaporator at pressure below 3 × 10^−6^ Torr.

### Extraction of interface trap density

The details of a subthreshold method to extract the interface trap density is explained elsewhere^33^. We used the equation of the drain current near the subthreshold regime:

$${I}_{D}={I}_{M}[1-\exp (-\frac{qm{V}_{D}}{nkT})]$$

where, $n=1+\frac{{C}_{d}+{C}_{it}}{{C}_{ox}}$, $m=1+\frac{{C}_{d}}{{C}_{ox}}$, *C*_*d*_ is the depletion capacitance, *C*_*it*_ is the interface capacitance, and *I*_*M*_ is the maximum drain current at a given gate voltage. Parameter *n* and ratio *m*/*n* can be obtained from the transfer characteristics of log *I*_*D*_ vs. *V*_*G*_ and the plot of −log(1 − *I*_*D*_/*I*_*M*_) vs. *V*_*D*_, respectively. The interface trap density as a function of energy level at a given gate voltage is determined as followed:

$${D}_{it}(E)=\frac{{C}_{it}(E)}{q}=\frac{{C}_{ox}}{q}(n-m)$$

where *q* is the electronic charge. The errors in the interface trap density were calculated from the standard deviation of the parameter *n* at different drain voltages and the standard deviation of the ratio *m/n*. It should be noted that the extraction of the interface trap density is not strongly affected by contact resistance because the extraction is performed in the subthreshold regime where the MoS_2_ channel resistance is much higher than the contact resistance.

### Transfer length method (TLM) measurement

For a more accurate analysis on the annealing effects, we extracted both contact resistance and mobility (excluding contact resistance) of the monolayer MoS_2_ FETs before and after the vacuum annealing using a typical TLM measurement^25, 26^, which is the standard method for the extraction of contact resistance in the FETs by fitting the on-state resistance as a function of channel length with the following equation:

$${R}_{ON}={R}_{CH}+{R}_{C}=\frac{L}{\mu {C}_{ox}({V}_{G}-{V}_{T})}+{R}_{C}$$

where *R*_*ON*_, *R*_*CH*_, and *R*_*C*_ are the on-state resistance, the channel resistance, and the contact resistance at a given gate voltage in excess of the threshold voltage, respectively, and all the resistances are in units of Ω mm, normalized by the channel width (*W*) to easily compare devices with different channel widths. From a plot of the on-state resistance (*R*_*ON*_) versus the channel length (*L*), the mobility *(μ*) and contact resistance (*R*_*C*_) can be extracted from its slope and y-intercept, respectively. We utilized a total 13 devices with at least three devices at a given channel length (*L*) and performed a linear regression to obtain the mobility *(μ*) and contact resistance (*R*_*C*_) as shown in Fig. 4b. The errors in the mobility and contact resistance were calculated from the standard deviation of the regression.

## Electronic supplementary material

Supplementary Information
