# Supplementary material for: Ambient effects on electrical characteristics of CVD-grown monolayer MoS2 field-effect transistors
Source: Sci Rep. 2017 Jun 22;7:4075. doi: 10.1038/s41598-017-04350-z (PMC5481332; doi:10.1038/s41598-017-04350-z)
Supplement: Supplementary file 1 — Supplementary Information [file 41598_2017_4350_MOESM1_ESM.pdf]

Supplementary Information

Ambient effects on electrical characteristics of CVD-grown monolayer MoS_2 field-effect transistors

Jae-Hyuk Ahn,[1,2] William M. Parkin,[1] Carl H. Naylor,[1] A. T. Charlie Johnson,[1] and Marija Drndić[1,a)]

[1] *Department of Physics and Astronomy, University of Pennsylvania, Philadelphia, Pennsylvania 19104, United States*

[2] *Department of Electronic Engineering, Kwangwoon University, Seoul 01897, South Korea*

[a)]Author to whom correspondence should be addressed. Electronic mail: drndic@physics.upenn.edu

Reproducibility of a pressure-dependent, non-monotonic change trend in threshold voltage

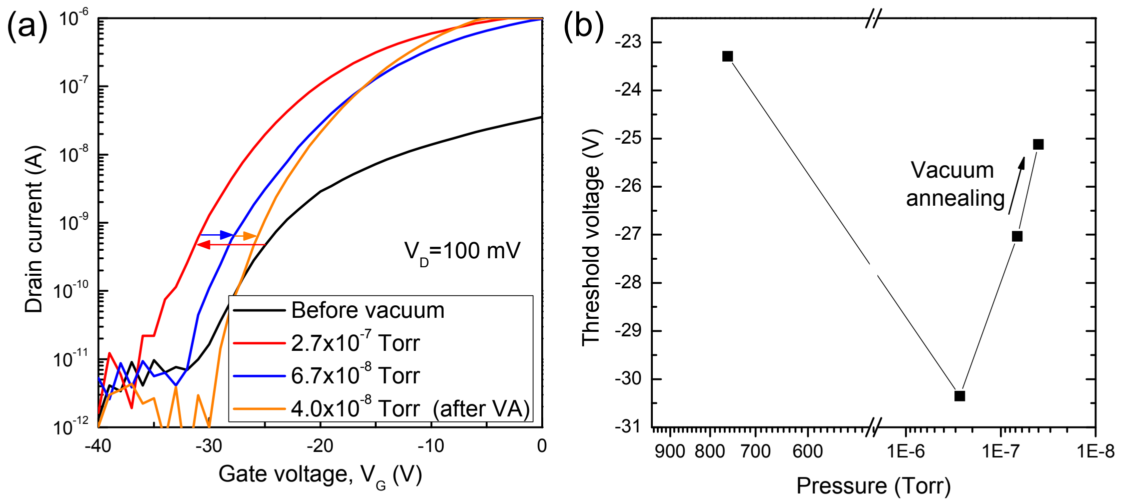

Figure S1. Dependence of pressure and vacuum annealing on threshold voltage (a) Transfer characteristics (I_D–V_G) of a monolayer MoS_2 FET with different ambient conditions. First, the I_D–V_G curve is shifted in the negative direction (from back to red) as the pressure is reduced to 2.7×10^{-7} Torr. Then, the curve is moved back to the positive side (from red to blue) with decreasing pressure to 6.7×10^{-8} Torr and shifted further in the positive direction (from blue to orange) by vacuum annealing (denoted as VA). (b) Threshold voltage with respect to the pressure, which is extracted from the transfer curves in Figure (a). The threshold voltage is reduced with decreasing pressure but increased again, showing a pressure-dependent, non-monotonic change in threshold voltage which is discussed in detail in the main text. In addition, further increase of the threshold voltage is observed after vacuum annealing.

Effects of vacuum annealing on electrical characteristics of MoS_2 FETs with different contact materials

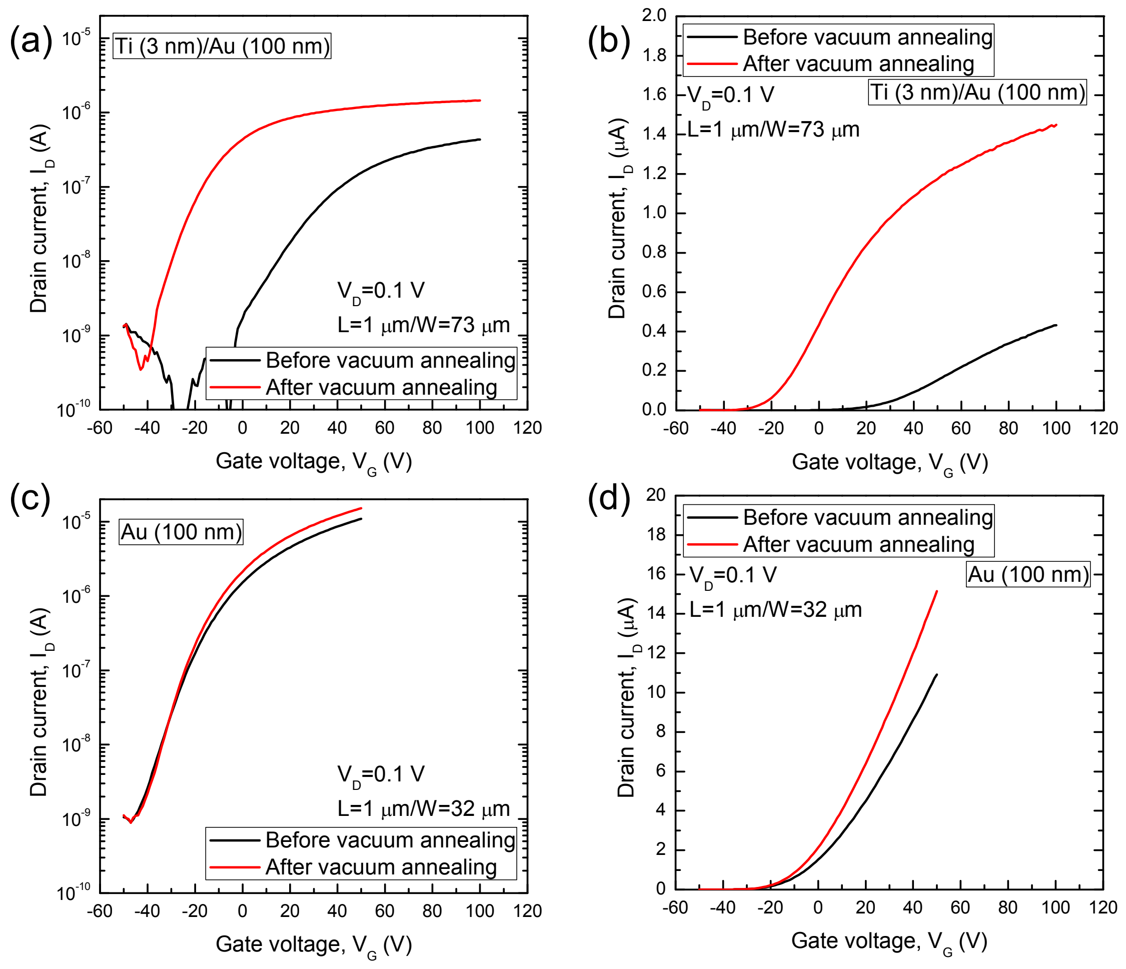

Figure S2. Electrical characteristics of monolayer MoS_2 FETs with different contact materials before and after vacuum annealing (400 K for ~12hr), measured at a pressure of 6.0×10^{-8} Torr. (a)-(b) a MoS_2 FET with Ti/Au contact (3 nm /100 nm). (c)-(d) a MoS_2 FET with Au contact (100 nm).

Mechanism of the non-monotonic dependence of threshold voltage on pressure

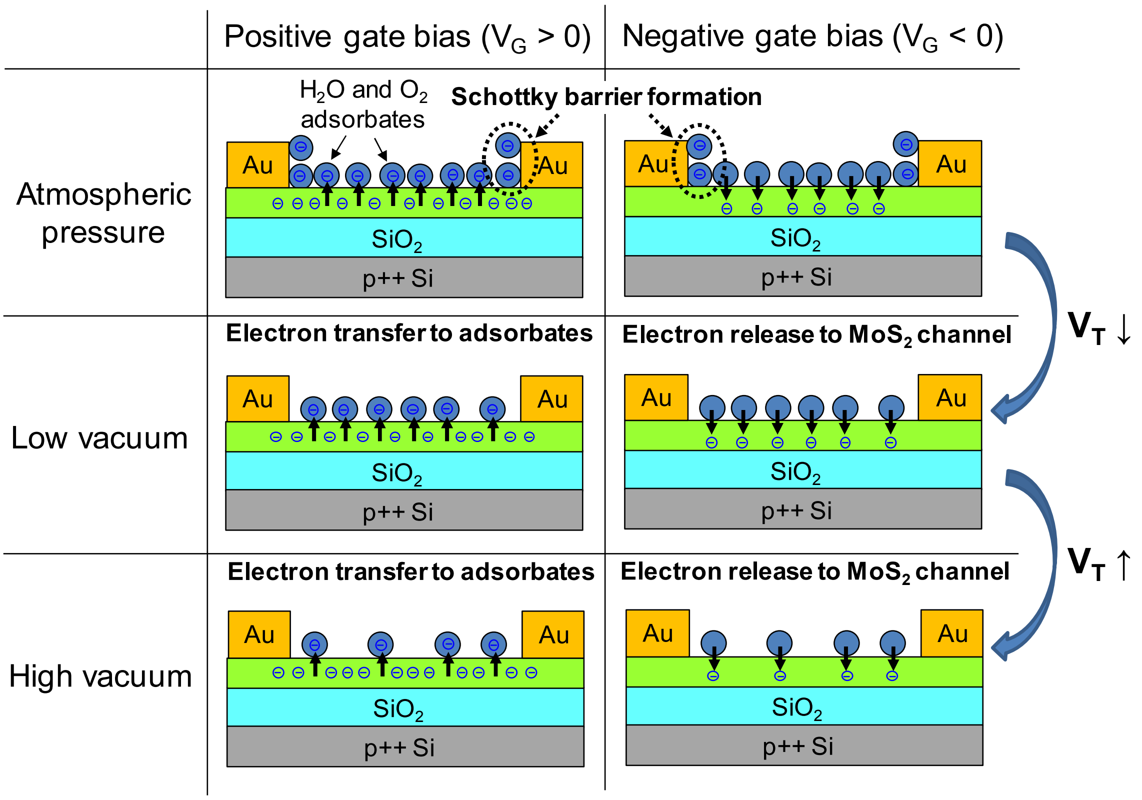

Figure S3. Schematics to explain the non-monotonic dependence of threshold voltage on pressure. As the water and oxygen molecules adsorbates near MoS_2-Au electrodes are desorbed, the contacts are changed from Schottky contacts to ohmic contacts, resulting in the the reduction of threshold voltage. Under high vacuum condition, the lower trap density reduces the number of carriers that can be released back to the channel when a negative gate voltage is applied, resulting in a decreased drain current and an increased threshold voltage.

Hysteresis in a top-gated monolayer MoS_2 FET

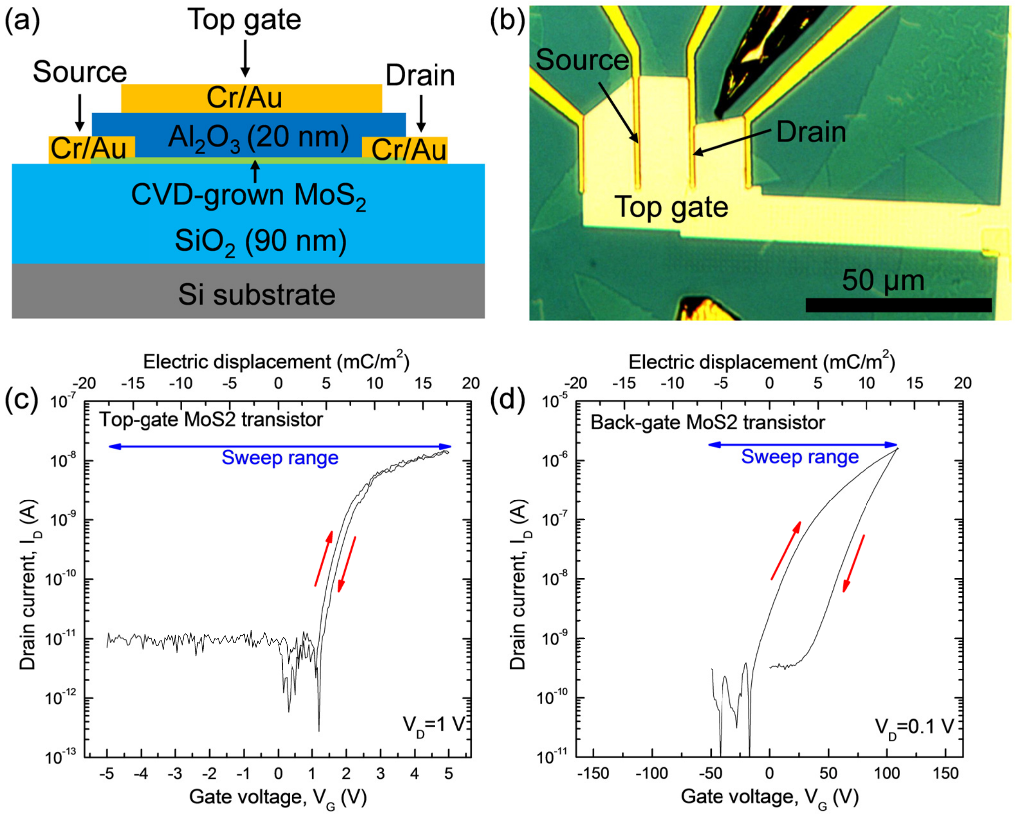

Figure S4. Comparison of the transfer characteristics (I_D–V_G) hysteresis between top-gated and back-gated MoS_2 FETs. Schematic (a) and optical image (b) of the top-gated MoS_2 FET. Transfer (I_D–V_G) characteristics of the top-gated MoS_2 FET (c) and the back-gated MoS_2 FET (d). The back-gated device had no passivation on top, leaving the top surface of the MoS2 channel exposed to ambient, unlike the top-gated device. The data for the back-gated device is from Figure 2(a) in the main text. For a fair comparison of hysteresis, the x-axis of the I_D–V_G curve is rescaled with the same electric displacement by considering the 20 nm-thick Al_2O_3 (ε_r=8.0) and 285 nm-thick SiO_2 (ε_r=3.9) for the top-gated and back-gated devices, respectively. Smaller hysteresis is observed in the top-gated MoS_2 FET even with the large sweep range.

Reduction of hysteresis after vacuum annealing

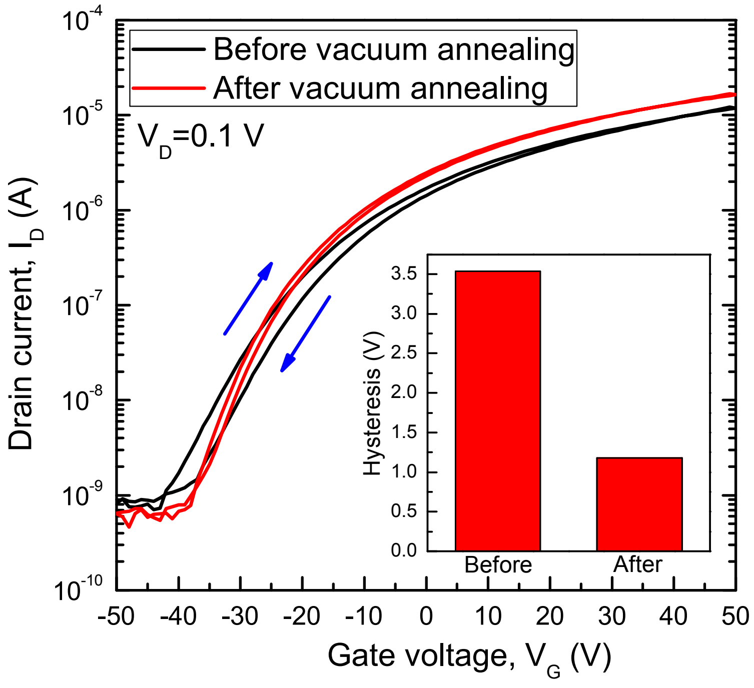

Figure S5. Comparison of the transfer characteristics (I_D–V_G) hysteresis before and after vacuum annealing (400 K for ~12hr), measured at a pressure of 6.0×10^{-8} Torr. Smaller hysteresis is shown after vacuum annealing.

Acceptor-type, shallow traps produced by the absorbed gas molecules

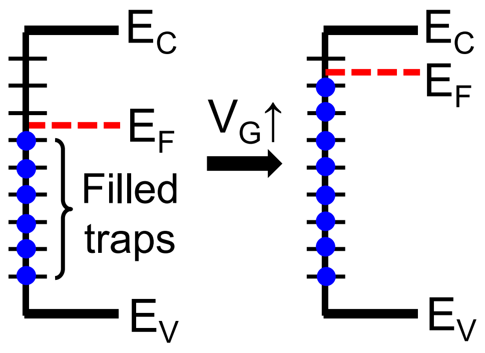

Figure S6. Schematic of energy band of the MoS_2 channel with absorbed water and oxygen molecules as interface traps filled with electrons below the Fermi level (E_F). The Fermi level is moved toward the conduction band with increasing gate voltage (V_G).

As shown in Fig. 2a in the main text, we observed the clockwise direction of the hysteresis in the I_D–V_G characteristics of the MoS_2 FETs: a lower threshold voltage in the forward gate sweep and a higher threshold voltage in the reverse gate sweep. From this experimental result, we can understand that the interface traps produced by the absorbed gas molecules are acceptor-type traps [1] that become negatively charged in a such way that the trap energy levels below the Fermi level (E_F) are filled by electrons (Fig. S5). The electrons in the MoS_2 channel can be transferred into the interface traps at positive gate bias, resulting in the increased threshold voltage of the reverse sweep. Then, the captured electrons can be released again to the MoS_2 channel at negative gate bias, resulting in the decreased threshold voltage for the forward sweep.

As the gate voltage is increased, the Fermi level (E_F) is moved toward the conduction band (E_C) and the interface traps at that energy level close to the conduction band is involved in the extraction of the interface trap density. Large difference of the interface trap density for the two ambient conditions with increasing gate voltage (V_G) as observed in Fig. 3b in the main text suggests that the interface traps are mainly located at shallow energy levels close to conduction band, which are typically called as shallow traps [1].

Effects of vacuum annealing on the contact resistance and mobility

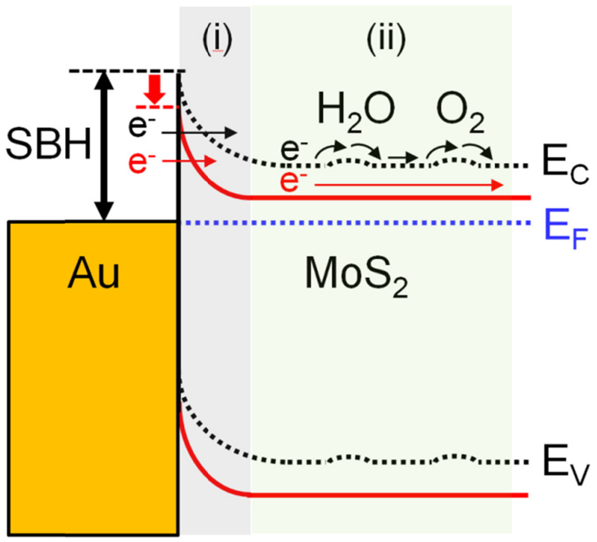

Figure S7. Energy band diagram along the MoS_2 channel and the Au contact before and after removal of water and oxygen molecules. When the absorbed gases that capture the electrons from the MoS_2 channel are detached by vacuum annealing, (i) the contact resistance is reduced due to a narrower tunneling width and reduced Schottky barrier height (SBH), and (ii) the mobility is improved due to a lower density of scattering centers and interface traps on the MoS_2 channel.

Based on a transfer length method (TLM) measurement, we observed the decreased MoS_2-metal contact resistance and the increased mobility of the MoS_2 channel after vacuum annealing (Fig. 4b in the main text). These experimental results can be further explained as followed.

As water and oxygen molecules are desorbed from the MoS_2-metal contacts by vacuum annealing, electrons that are initially transferred to the molecules in the on-state regime are now returned to the MoS_2 channel, inducing channel doping. In the energy band diagram as depicted in Fig. S7, the Fermi level (E_F) is moved up toward the conduction band (E_C) after removal of water and oxygen molecules due to channel doping. This leads to the reduction of the Schottky barrier height (SBH) defined as the difference between the interfacial conduction band and the Fermi level. Consequently, a lower contact resistance is expected because of a narrower tunneling width and reduced Schottky barrier height as shown in Fig. S7. In addition, the mobility is increased due to a lower density of molecules on the MoS_2 channel surface which serve as scattering centers or interface traps (Fig. S7).

References

[1] Sze, S. M. & Ng, K. K. *Semiconductor Devices: Physics and Technology.* (Wiley, 2002).
